# Real world experience with faricimab in switched neovascular AMD and evaluation of reloading versus interval matching regimes

**DOI:** 10.1038/s41433-026-04686-9

**Published:** 2026-07-03

**Authors:** Ffion E. Brown, Madhuparna Mitra

**Affiliations:** Aneurin Bevan University Health Board, Royal Gwent Hospital, Newport, UK

**Keywords:** Drug therapy, Health services

## Abstract

**Background:**

A subset of eyes with neovascular AMD experience sub-optimal response to anti-VEGF therapy resulting in frequent injections. Faricimab has been used in this cohort due to reputed increased durability. Dosing strategies when switching vary, particularly regarding the need for a reloading phase. Our aim is to share our real-world experience of switching to faricimab with a clinician-directed dosing strategy.

**Methods:**

Retrospective analysis of eyes with neovascular AMD switched to faricimab from a previous anti-VEGF agent in Aneurin Bevan University Health Board, South Wales. Outcomes were evaluated at one year.

**Results:**

173 eyes from 134 patients were analysed. Mean visual acuity remained stable at one year (–0.1 letters). There was a statistically significant reduction in central subfield thickness of 30.0 μm (95% CI − 41.1 to −18.9; *p* < 0.001) and an increase in injection interval of 2 weeks (95% CI 1.5 to 2.4; *p* < 0.001). Within these parameters, the ‘Reloaded’ subgroup (*n* = 102) showed no clear advantage over the ‘Not reloaded’ (interval matched ± 2 weeks) subgroup (*n* = 71). The ‘Reloaded’ subgroup had received fewer prior injections and had shorter baseline injection intervals before switching.

**Conclusions:**

Switching to faricimab was associated with stable vision, reduced macular oedema and increased injection intervals at one year. A clinician-directed dosing strategy at switching yielded comparable outcomes between subgroups, suggesting a reloading phase may not be required for all eyes. This has potential benefits for reducing demand on service capacity.

## Introduction

The prevalence of age-related macular degeneration is increasing with an aging population and is predicted to affect 288 million people worldwide by 2040 [1]. Neovascular age-related macular degeneration (nAMD) accounts for 4–7% of cases but over 90% of those with severe visual loss [2]. Treatment for nAMD with anti-vascular endothelial growth factor (anti-VEGF) injections has successfully halved the incidence of blindness [2]. A subset of eyes with nAMD have a suboptimal response to anti-VEGF therapy, hence requiring more frequent injections. This is both a burden for the patient and a strain on hospital services [3].

It has been well reported that an increased demand in a limited capacity service is a significant issue for UK ophthalmology, and particularly nAMD services [4]. Delayed follow up appointments can lead to complications, resulting in detrimental health and economic impacts to the patient, healthcare service and society. One solution is the development of anti-VEGF treatments with increased durability, hence requiring less frequent clinic attendances per patient [4].

Faricimab is a bispecific monoclonal antibody which targets both VEGF-A and angiopoetin-2. Phase III studies in treatment naive patients demonstrated stable vision, rapidly improved anatomical markers and good durability with injection interval extension up to 16 weeks [5, 6]. Results from real world data have been comparable, including benefit from switching patients with inadequate response to other anti-VEGF agents [7]. While Phase III trials recommended four 4-weekly loading injections, many studies of switched patients utilise a ‘Reloading’ regime of three or four 4-weekly injections of faricimab before extending the treatment interval [8–11]. The posology of faricimab now reflects this, suggesting dosing intervals at clinician discretion when switching [12]. However, the necessity of a reloading phase has not been fully explored and is debated amongst retina specialists [13].

Our aim was to share our real-world experience of switching eyes with nAMD to faricimab in our health board in South Wales. This included a subset analysis to observe if there was any clinical benefit in ‘Reloading’ eyes at the point of switch compared to interval matching ±2 weeks. Additionally, we examined eyes on 8-weekly or longer injection intervals prior to switching to identify if there was any merit in switching this cohort in terms of clinical outcome and further extension of treatment intervals.

## Methods

A retrospective data analysis of eyes with nAMD switched to faricimab from a previous anti-VEGF agent. Data was collected from three injections centres across Aneurin Bevan University Health Board, South Wales. Each eye was followed up for a 1-year period.

**Fig. 1:**
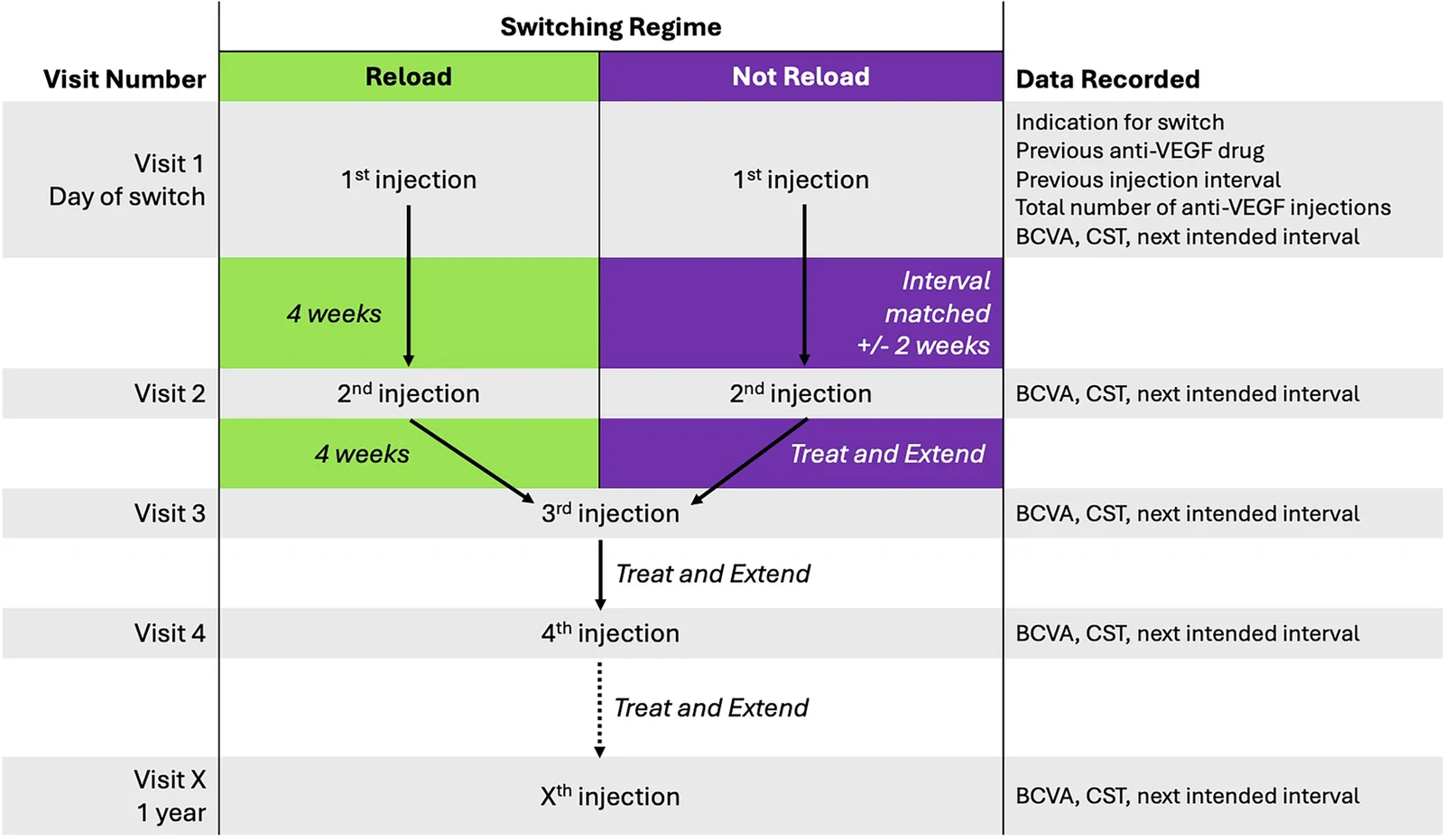
Local pathways for switching to faricimab from a previous anti-VEGF drug. Patients were switched using the ‘Reload’ (green) or ‘Not reload’ (purple) regimes. Data was recorded retrospectively from the day of switch (Visit 1) until 1 year (Visit X).

The decision to switch to faricimab was at the discretion of the individual clinician guided by patient-specific factors. At the day of switch, termed Visit 1, baseline data was recorded including demographic details, indication for switch, previous anti-VEGF treatment history, visual acuity and optical coherence tomography (OCT) findings. Individual clinicians also decided at Visit 1 whether to ‘Reload’ patients; meaning three 4-weekly injections before continuing along a standard ‘treat and extend’ pathway. This differs from the initial recommended drug posology from Phase 3 trials conducted on treatment naïve patients which advised a loading regime of four times 4-weekly injections [6]. The decision to reload with three rather than four injections was a local departmental decision made to practically facilitate switching patients to faricimab in a limited capacity service. It is also in keeping with the suggestion for switching from the TRUKEE study which investigated real world data for patients with AMD [5]. If clinicians decided to ‘Not reload’, the patient was interval matched with their treatment interval on the previous anti-VEGF agent ±2 weeks (Fig. 1).

Eyes were excluded if treatment was stopped or interrupted for a period of three months or more within the one-year follow-up period. No other exclusion criterion was applied to best reflect our real-world outcomes.

Data from each visit was collected from patient case notes and OCT imaging. Best corrected visual acuity (BCVA) was measured using EDTRS letters. The normality assumption of data was evaluated by visual inspection of histograms and the distribution appeared approximately symmetric. Independent and paired sample *t* tests were performed on numerical outcomes with statistical significance deemed *p* < 0.05. For independent-samples comparisons, the homogeneity of variance was assessed using Levene’s test. When the assumption was breached, Welch’s t-test was applied. The study was registered as a service evaluation with Aneurin Bevan University Health Board and did not require ethical approval as per NHS Research Ethics Committee guidelines for Wales.

## Results

**Fig. 2:**
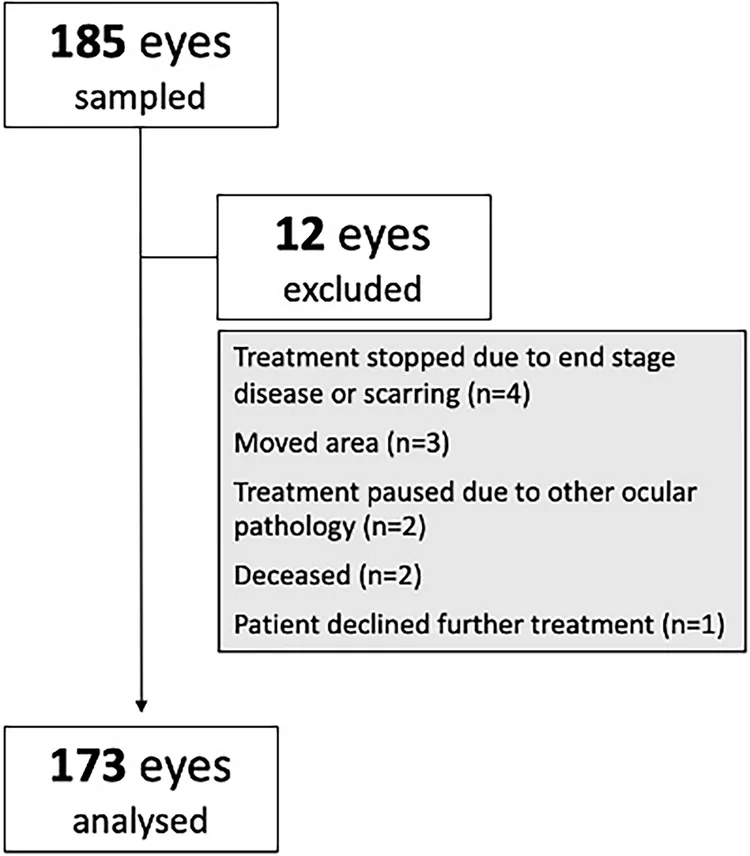
Patient inclusion flow chart.

One hundred and eighty-five eyes were sampled with 12 eyes excluded, leaving 173 eyes from 134 patients included for analysis (Fig. 2). This cohort had a mean age of 78 years with a male: female ratio of 1: 1.4. Mean baseline BCVA was 62 letters and mean central subfield thickness (CST) 276μm. Eyes had received a mean of 23 previous anti-VEGF injections and the majority (81%) were switched from aflibercept.

Of this cohort, 102 eyes (59%) were ‘Reloaded’ and 71 eyes (41%) were ‘Not reloaded’. These subgroups showed no statistically significant baseline differences in demographics, including sex (*p* = 0.32), age (*p* = 0.98), baseline vision (*p* = 0.74) and baseline CST (*p* = 0.32). The ‘Not reloaded’ cohort differed to the ‘Reloaded’ cohort through a higher number of previous anti-VEGF injections (*p* = 0.002) and a longer previous injection interval prior to switch (*p* = 0.002) (Table 1).

**Table 1:** Demographic and baseline data for the total cohort and ‘Reloaded’ and ‘Not reloaded’ subgroups.

	Total cohort	Reloaded (*n* = 102)	Not reloaded (*n* = 71)
	(*n* = 173)		
Age (years) (mean, range)	78 (51–96)	78 (55–93)	78 (51–96)
Baseline BCVA (letters) (mean, range)	62 (20–89)	62 (20–89)	61 (27–84)
Baseline CST (µm) (mean)	276 (132–689)	281 (135–689)	269 (132–458)
Number of anti-VEGF injections prior to switch (mean)	23	20	27
Type of anti VEGF (%)			
Aflibercept	81	93	63
Ranibizumab	14	4	28
Ongavia	5	3	9
Injection interval prior to switch (weeks) (mean)	6.6	6.3	7

Indications for switching to faricimab could be multiple; 92% of eyes were switched due to persistent macular oedema, 45% of eyes were unable to extend the treatment interval, 28% of eyes had reduced visual acuity and 3% were second eyes with no other indication.

During the one year follow up period, eyes had a mean of 7.68 injections; 8.08 in the ‘Reloaded’ subgroup and 7.10 in the ‘Not reloaded’ subgroup.

### Vision

**Fig. 3:**
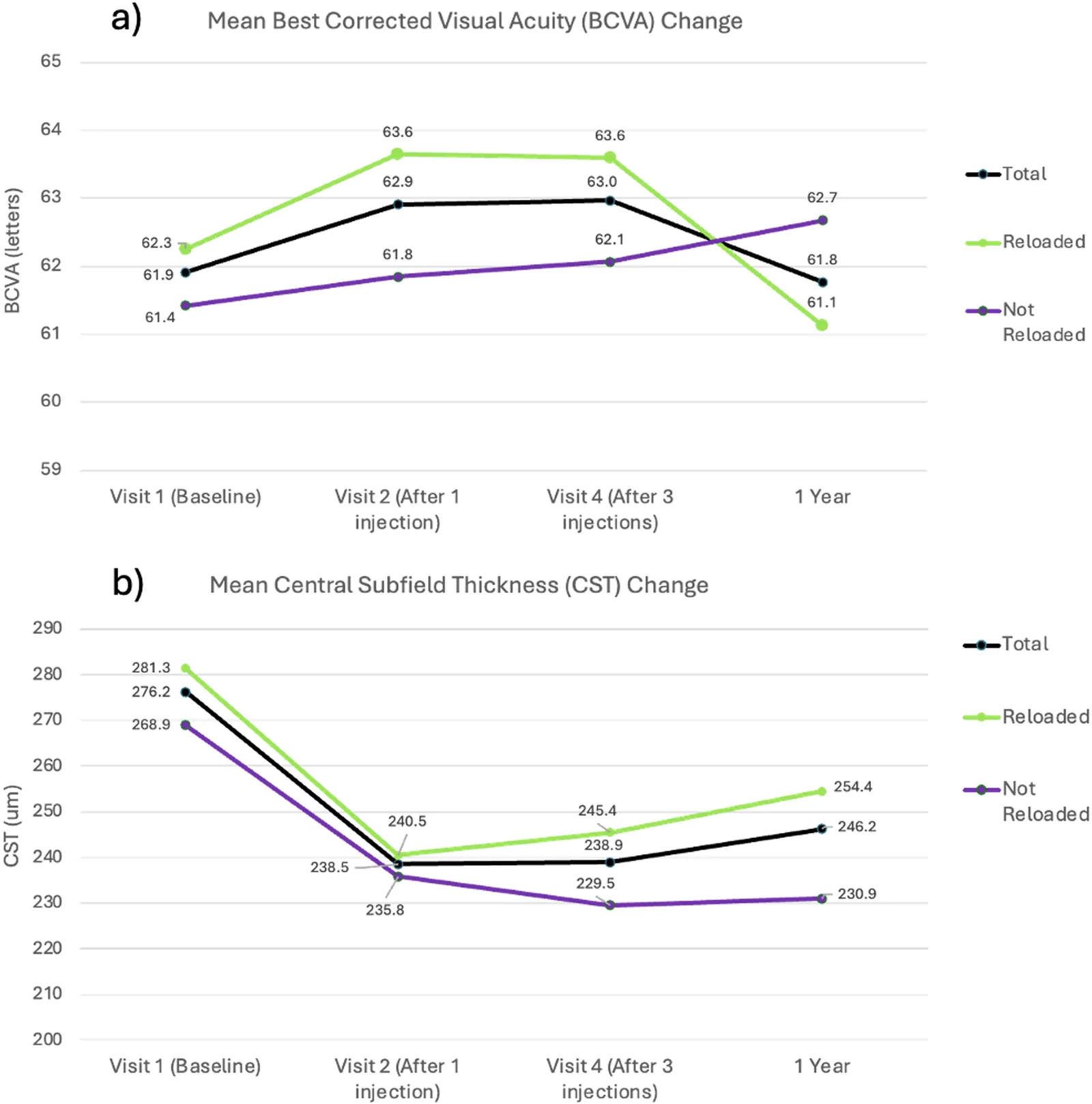
Longitudinal changes in visual acuity and macular oedema in eyes following switching to faricimab. **a** Mean change in best corrected visual acuity (BCVA). **b** Mean change in central subfield thickness (CST). Results shown for the total population (black), ‘Reloaded’ subgroup (green) and ‘Not reloaded’ subgroup (purple).

BCVA remained stable at one year post switch with a mean change of –0.1 letters. For the ‘Reloaded’ and ‘Not reloaded’ cohorts the mean change was –1.1 letters (95% CI − 3.3 to 1.1; *p* = 0.31) and +1.3 letters (95% CI − 0.76 to 3.27; *p* = 0.22), respectively (Fig. 3a). This difference was not statistically significant and unlikely to have any functional significance to the patient. In the subgroup of 28% eyes (*n* = 49) switched due to reduced visual acuity, BCVA changed by +0.7 letters at one year.

### Macular oedema

For the total population, there was a statistically significant reduction in CST of 30.0μm at one year (95% CI − 41.1 to −18.9; *p* < 0.001). The ‘Reloaded’ subgroup had a reduction of 25.2μm (95% CI − 40.3 to −10.1; *p* = 0.0013) and the ‘Not reloaded’ by 36.9μm (95% CI − 53.5 to −20.4; *p* < 0.001) (Fig. 3b).

At Visit 1, 8% of the total population were reported to have a dry macula. This increased to 46% after only one faricimab injection and remained at 30% at one year. A quarter of the total population had both sub-retinal and intra-retinal fluid at Visit 1, which reduced to 9% at one year.

### Injection interval

At baseline, 60% of the total population were on 4- or 6-weekly injection intervals. This almost halved to 32% at one year. Those on 12+ weekly injection intervals increased from <1% at baseline to 21%. Overall, the mean injection interval for the total population increased from 6.6 weeks to 8.6 weeks at one year (95% CI 1.5–2.4; *p* < 0.001).

**Fig. 4:**
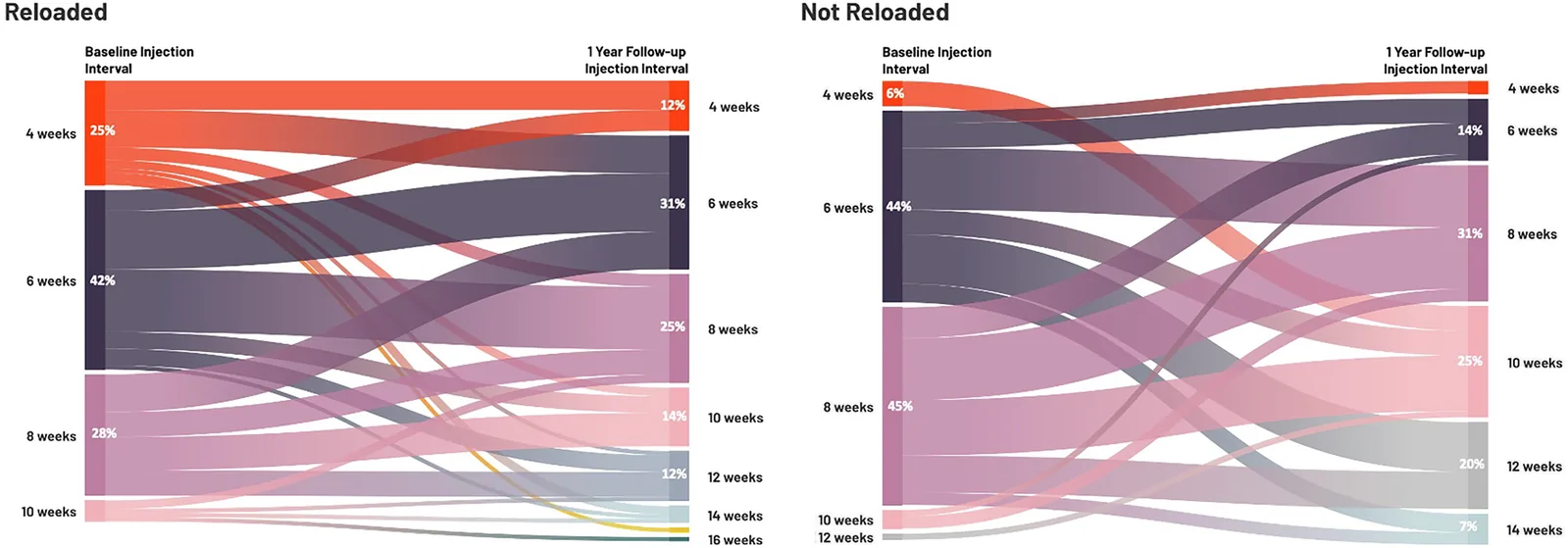
Sankey diagram showing the change in injection interval at one year following switching to faricimab. The left panel is the ‘Reloaded’ subgroup and the right panel the ‘Not reloaded’ subgroup.

The ‘Reloaded’ subgroup had 67% on 4- or 6-weekly injection intervals at baseline which reduced to 43% at one year. The ‘Not reloaded’ subgroup had a lower starting baseline of 50% on 4- or 6-weekly injection intervals which further decreased to 17% at one year. Both subgroups had a statistically significant increase in injection interval at one year by 1.8 weeks (95% CI 1.2–2.4; *p* < 0.001) and 2.3 weeks (95% CI 1.6–3.0; *p* < 0.001) for ‘Reloaded’ and ‘Not reloaded’ respectively (Fig. 4).

### ‘8+ Weekers’ subgroup

Seventy eyes were on 8–12 weekly injection intervals prior to switching to faricimab. Baseline demographics were similar to the total cohort (Supplementary Table 1). At one year, BCVA and CST had changed by +0.2 letters and –32.6 μm, respectively. Injection interval increased from a mean of 8.3 weeks at Visit 1 to 9.2 weeks at one year (Supplementary Fig. 1.). This increase of 0.9 weeks was statistically significant (95% CI 0.33–1.50; *p* = 0.0028).

### Safety

During the follow up period, there was one report of a possible side effect to faricimab. The patient developed raised intraocular pressure following their fifth faricimab injection, requiring paracentesis and medical management. Mild anterior chamber inflammation was recorded with fine keratic precipitates. Previously, the patient had similar intraocular pressure spikes with other anti-VEGF agents. Treatment was paused whilst a glaucoma opinion was sought, hence the eye was excluded from analysis. Following urgent glaucoma surgery, the patient has restarted faricimab.

## Discussion

Our results contribute to the literature on the safety and efficacy of faricimab in eyes which have previously failed to adequately respond to other anti-VEGF agents. BCVA remained stable at one year, even in the subset that were switched to faricimab due to reducing vision. There was a statistically significant reduction in CST and an increase in injection interval at one year. Eyes which were ‘Reloaded’ had differing treatment history, with shorter previous injection intervals, suggesting more active disease compared to the ‘Not reloaded’ subgroup. Within the limitation of this ‘clinician discretion’ approach to reloading, we found comparable outcomes between eyes reloaded with three 4-weekly injections compared to eyes interval matched ±2 weeks. Furthermore, there was also a modest increase in injection interval when switching those already on longer (8+ weekly) injection intervals at baseline.

### Anatomy and function

Despite improved anatomical markers following switching to faricimab, this did not correlate with any functional improvement in BVCA. This is consistent with findings of other reports when switching eyes with a previous sub-optimal anti-VEGF response [3, 11, 13, 14]. The chronicity and severity of disease in this cohort implies underlying photoreceptor degeneration which will limit vision improvement [13].

### Reloading

Clinical evidence for the use of anti-VEGF agents in treatment naïve patients strongly supports a loading phase to reduce the time taken to reach therapeutic levels [15]. Hence, many studies assessing eyes switched to faricimab have also included a loading phase, presumably to maximise the benefit of the additional angiopoietin-2 blockade effect [8–11]. The TRUCKEE study, a large retrospective study of eyes with nAMD treated with faricimab, also recommended a loading phase when switching. They suggest three 4-weekly injections or maintaining the current injection interval for three injections before extending. Their rationale was that multiple doses were required before the maximum benefit of faricimab was observed [5]. However, results were only reported after one and three injections of faricimab with no direct comparison between those who were interval matched or reloaded.

Other studies have also used interval matched dosing at switch. Hitikini et al. switched patients with nAMD directly onto a treat and extend regime. In their cohort of 48 eyes, they demonstrated stable BCVA and an injection interval extension of 3.7 weeks at 6 months [16]. Borchert et al. took a pragmatic approach with when switching, with 25% of their cohort of 107 eyes reloading with four 4-weekly injections and 75% extending more rapidly [13]. However, there was no direct comparative analysis between these subgroups reported.

This real-world study observed outcomes of eyes that underwent a reloading phase compared with those which were interval-matched when switching to faricimab. The choice of dosing regimen at switching was clinician-led and patient-specific, reflecting the updated posology of faricimab. Within this framework, eyes that were ‘Not-reloaded’ demonstrated similar favourable outcomes as to those which were ‘Reloaded’. This suggests that a reloading phase may not be a requirement in all cases to observe the benefits of switching to faricimab. This is important as reloading may be a barrier to switching patients both for service capacity and patient choice due to the burden of more frequent injections initially.

### Generalisability

Whilst our data is from a single UK health board, our total population results mirror those reported from other UK centres when switching eyes with previous sub-optimal anti-VEGF response to faricimab [3, 13]. However, dosing regimens when switching vary between health boards with most across South West and Wales opting for a three or four 4-weekly reloading phase [17]. Due to the updated posology for faricimab in switched eyes [12] we anticipate more health boards will move towards a clinician-led, patient-specific approach to treatment intervals when switching.

### Capacity

From the assumption that eyes switched to faricimab were able to extend the injection interval by 2 weeks, the potential impact on service capacity can be modelled. Simply, we can assume a constant injection interval for the year prior and post switch. For this cohort of 173 eyes, the mean baseline injection interval of 6.6 weeks equates to 1363 injections per year. At the mean 1-year injection interval of 8.6 weeks, this reduces to 1046 injections per year. For Aneurin Bevan University Health Board, where there are 22 injections per clinic, this is a reduction in 14 clinics per year. This model, however, overestimates the impact of switching in the first year as injection interval extension occurs gradually. A linear model provides a more conservative approach by assuming a gradual injection increase rather than an immediate step change. For 173 eyes, modelling a linear increase from 6.6 to 8.6 weeks relates to a reduction in 180 injections per year, correlating to 8 clinics. Extrapolating these results to the large number of patients receiving intravitreal injections for nAMD has the potential to have significant impact on increasing service capacity. However, a larger study with a longer follow up period would be required to confirm these projections.

Furthermore, switching to faricimab without always requiring reloading further reduces the burden on injection appointments. Additionally, through demonstrating a modest increase in injection interval of those already on 8+ weekly injection intervals, there is likely benefit to broadening the criteria of whom may benefit from switching to faricimab away from predominately those on 4- or 6- weekly injection intervals.

### Safety

In our study, faricimab was overall safe. The risk of uveitis in association with faricimab is estimated at 1:100 to 1:1000 from clinical trials and our study reflects this [18]. Other studies suggest this risk is comparable with other anti-VEGF agents [19].

### Limitations

There are several limitations of this study. Firstly, the previous treatment history differed between the ‘Reloaded’ and ‘Not reloaded’ subgroups. Eyes in the ‘Reloaded’ subgroup had shorter previous injection intervals prior to switch, suggesting more active or severe disease compared with the ‘Not reloaded’ subgroup. This likely influenced the clinician’s decision to reload these eyes. While this reflects pragmatic real-world practice, it introduces differences between the subgroups which must be considered when making direct comparisons. These differences may explain why the ‘Not reloaded’ subgroup marginally outperformed the ‘Reloaded’ subgroup with a larger reduction in CST and slightly longer injection interval at one year. Furthermore, by nature of not reloading in the first three months, the ‘Not reloaded’ group had a longer period in which to extend their treatment intervals within the one year follow up period. Regardless, within these parameters, both subgroups independently demonstrated statistically significant benefits from switching to faricimab. Further studies with a prospective randomised design would likely be beneficial in validating our retrospective findings, especially in eyes with more active or severe disease.

Secondly, during this study, there was no control group for direct comparison to how eyes would have responded to another anti-VEGF agent across the same period. It is the expected natural course of treatment that the injection interval will extend with time [15]. However, our population had a mean of 23 previous anti-VEGF injections and still had not extended past a mean of 6.6 weeks despite this. Therefore, previous anti-VEGF agents had already been trialled on average for approximately 3 years and the eye considered to have a suboptimal response. In our cohort, therefore, it is likely the switch to faricimab explains the anatomical improvements and improved durability of treatment rather than purely being a consequence of the time course of treatment.

Furthermore, the injection intervals recorded in this study are the intended treatment intervals and the actual interval may have differed due to external factors such as patients not attending or cancelling appointments.

### Conclusion

Eyes with nAMD switched to faricimab demonstrated stable vision, reduced macular oedema and an increased injection interval at one year follow up.

Clinician discretion was used to determine dosing regimen at switching either with a formal reloading phase of three 4-weekly injections or interval matched ±2 weeks. Eyes with fewer previous injections and shorter injection intervals at baseline tended to be reloaded. Our real-world data found similar outcomes between the subgroups, suggesting a clinician-led approach to dosing at switching is appropriate, as a reloading phase may not be required for all eyes to experience the benefit of switching. Our outcomes support an individualised, patient-centred approach in line with the updated posology of faricimab at switching. Prospective studies are needed to further assess the benefit of reloading, especially in eyes with more active disease.

Overall, our findings indicate that a clinician-discretion approach to dosing when switching eyes to faricimab is effective. There was also a modest benefit in increasing injection intervals in eyes already on 8+ weekly injection intervals, suggesting the criteria for switching has scope to be broadened. Together, these findings have potential benefits in reducing appointment burden and hence demand on service capacity. Supplemental material is available at Eye’s website.

#### What was known before:

- Switching eyes with nAMD, who are not responding to other-anti-VEGF agents, to faricimab is safe and effective.
- Faricimab has increased durability compared to other anti-VEGF agents, allowing for increased injection intervals.
- Many studies utilise a ‘reloading’ regime of three or four 4-weekly injections of faricimab when switching before extending the treatment interval.

#### What this study adds:

- We switched eyes to faricimab using a clinician-led approach, enabling evaluation of outcomes from eyes that were reloaded compared to interval matched ±2 weeks.
- In relation to change in vision, macular oedema and injection interval, our clinician-led dosing strategy at switching yielded comparable outcomes between the subgroups.
- Switching eyes to faricimab may not always require a reloading phase which, alongside its increased durability, has potential benefits in reducing demand on service capacity.

## Supplementary information

Supplementary Legends

Supplementary Figure 1

Supplementary Table 1

## Data Availability

The datasets generated and analysed during this study are available from the corresponding author on reasonable request.
