# Supplementary material for: Real world experience with faricimab in switched neovascular AMD and evaluation of reloading versus interval matching regimes
Source: Eye (Lond). 2026 Jul 3;40(14):2199–205. doi: 10.1038/s41433-026-04686-9 (PMC13601533; doi:10.1038/s41433-026-04686-9)
Supplement: Supplementary file 1 [file 41433_2026_4686_MOESM1_ESM.docx]

**Supplementary Legends**

Supplementary Table 1. Demographic and baseline data for the ‘8+ Weekers’ subgroup

Supplementary Figure 1. Sankey diagram showing the injection interval prior to switching to faricimab (Baseline) and at one year follow up for the ‘8+ Weekers’ subgroup.
